# On Imbalance of Impulse Control and Sensation Seeking and Adolescent Risk: An Intra-individual Developmental Test of the Dual Systems and Maturational Imbalance Models

**DOI:** 10.1007/s10964-021-01419-x

**Published:** 2021-03-20

**Authors:** Wim Meeus, Wilma Vollebergh, Susan Branje, Elisabetta Crocetti, Johan Ormel, Rens van de Schoot, Eveline A. Crone, Andrik Becht

**Affiliations:** 1 Utrecht University, Utrecht, The Netherlands; 2 Alma Mater Studiorum Università di Bologna, Bologna, Italy; 3 University of Groningen, University Medical Centre Groningen, Groningen, The Netherlands; 4 Erasmus University, Rotterdam, The Netherlands

**Keywords:** Dual systems models, Imbalance hypothesis, Developmental trajectories, Substance use, Adolescent risk

## Abstract

Heterogeneity in development of imbalance between impulse control and sensation seeking has not been studied until now. The present study scrutinized this heterogeneity and the link between imbalance and adolescent risk. Seven-wave data of 7,558 youth (50.71% males; age range from 12/13 until 24/25) were used. Three developmental trajectories were identified. The first trajectory, “sensation seeking to balanced sensation seeking”, included participants with a higher level of sensation seeking than impulse control across all ages. The second trajectory, “moderate dominant control”, included participants showing moderate and increasing impulse control relative to sensation seeking across all ages. The third trajectory, “strong late dominant control”, included participants showing the highest level of impulse control which was about as strong as sensation seeking from early to middle adolescence and became substantially stronger from late adolescence to early adulthood. Although the systematic increase of impulse control in all subgroups is in line with both models, neither of these combined trajectories of control and sensation seeking was predicted by the Dual Systems Model or the Maturational Imbalance Model. Consistent with both models the “sensation seeking to balanced sensation seeking” trajectory showed the highest level of substance use. It can be concluded that, even though both theories adequately predict the link between imbalance and risk, neither the Dual Systems Model nor the Maturational Imbalance Model correctly predict the heterogeneity in development of imbalance between impulse control and sensation seeking.

## Introduction

**Fig. 1:**
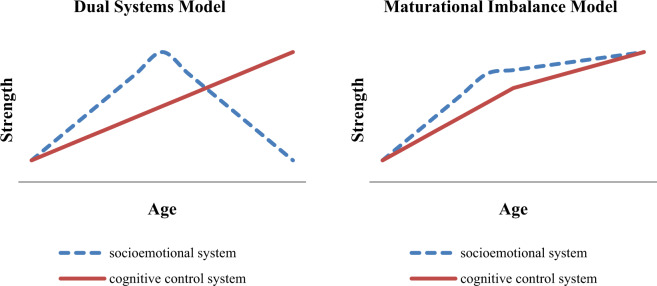
Alternative theoretical models of the differential development of the socioemotional and cognitive control system between ages 12 and 25

The Dual Systems Model (Steinberg, 2008) and the Maturational Imbalance Model (Casey et al., 2008) assume that adolescent risk-taking results from the temporary imbalance between two neurobiological systems: the subcortical socioemotional system that is responsive to emotion, reward and novelty, and the prefrontal cognitive control system that guides impulse control, planning and decision making. A key assumption of the Dual Systems Model and the Maturational Imbalance Model is that the socioemotional system develops faster in adolescence than the cognitive control system. As a result, especially middle adolescents (ages 14 until 17) are presumed to be vulnerable for high risk taking. Both models predict that at the end of adolescence cognitive control has matured and is more highly developed than the tendency to seek novelty and reward. As can be seen in Fig. 1 the models differ in their prediction of the developmental change of socioemotional reactivity and cognitive control in late adolescence and early adulthood. The Dual Systems Model predicts that cognitive control will be more highly developed than socioemotional reactivity whereas the Maturational Imbalance Model assumes that both behavioral tendencies will be about as strong (i.e., in balance). Therefore, the current study puts forth the proposition that the key assumption of the Dual Systems Model and the Maturational Imbalance Model is that the balance of socioemotional reactivity and cognitive control changes within individuals during adolescence. This key assumption of the Dual Systems Model and the Maturational Imbalance Model has not been properly tested until now. This calls for a longitudinal design that allows studying intra-individual change of the configuration of cognitive control and socioemotional reactivity, as well as heterogeneity of this process of intra-individual change. The present study aims to provide this longitudinal test of both models. A longitudinal person-centered approach will be adopted to test whether the balance between socioemotional reactivity and cognitive control develops as predicted by the Dual Systems Model and the Maturational Imbalance Model. The link between (im)balance and risk behavior from early adolescence until early adulthood will also be studied.

Choosing this approach makes it possible to meet a couple of critiques on the Dual Systems Model and the Maturational Imbalance Model. First, most of the Dual Systems Model and the Maturational Imbalance Model studies are cross-sectional and therefore cannot be used to draw inferences on the development of cognitive control and socioemotional reactivity. The approach of the present study is longitudinal and therefore makes it possible to describe the development of cognitive control and socioemotional reactivity across adolescence and early adulthood. Second, most of the studies seek verification by focusing on supportive evidence instead of falsification by proposing precise and falsifiable predictions (Pfeifer & Allen, 2016), for instance that in one group cognitive control overrides socioemotional reactivity while, in another group, cognitive control and socioemotional reactivity gain equal strength at the end of adolescence. Since the present study posits that the key assumption of the Dual Systems Model and the Maturational Imbalance Model is that the balance of socioemotional reactivity and cognitive control changes within individuals across time, it is possible to formulate specific hypotheses on this pattern of change. Before reaching these hypotheses, first an overview of longitudinal studies on the development of cognitive control and socioemotional reactivity and adolescent risk taking will be presented.

### Dual Systems, Developmental Issues, and Adolescent Risk Taking

#### Development of cognitive control and socioemotional reactivity

Do longitudinal studies support the assumptions of the Dual Systems Model and the Maturational Imbalance Model on the growth of cognitive control? Three types of longitudinal studies which typically analyze the development of cognitive control have been conducted: self-report (Ashenhurst et al., 2015; Atherton et al., 2020; Chaku & Hoyt, 2019; Collado et al., 2014; Harden & Tucker-Drob, 2011; Kasen et al., 2011; Khurana et al., 2018; Littlefield et al., 2009; Lydon-Staley & Geier, 2018; Pedersen et al., 2012; Shulman et al., 2015), behavioral (Achterberg et al., 2016; Almy et al., 2018; Anokhin et al., 2011; Bezdijan et al., 2014; Dougherty et al., 2015; Fosco et al., 2019; Khurana et al., 2018), and neurocognitive (Cope et al., 2020; Peters et al., 2016, Simmonds et al., 2017) studies. The self-report and behavioral studies used various measures: personality scales, temperament questionnaires, and gambling tasks, respectively. The neurocognitive studies primarily used go/no-go tasks, working memory tasks in combination with functional Magnetic Resonance Imaging (fMRI) data of various brain regions of interest. With a single exception (Collado et al., 2014) the studies show growth of cognitive control across adolescence and early adulthood. These findings offer partial support to both the Dual Systems Model and the Maturational Imbalance Model.

The Dual Systems Model assumes a curvilinear development of socioemotional reactivity, whereas the Maturational Imbalance Model assumes a strong increase in the first half of adolescence and a flattening thereafter (see Fig. 1). Three types of longitudinal studies into the development of the socioemotional system have been conducted: self-report (Ashenhurst et al., 2015; Collado et al., 2014; Crawford et al., 2003; Harden & Tucker-Drob, 2011; Khurana et al., 2018; Lydon-Staley & Geier, 2018; Lynne-Landsman et al., 2011; MacPherson et al., 2010; Pedersen et al., 2012; Romer & Hennessy, 2007; Shulman et al., 2015), behavioral (Khurana et al., 2018), and neurocognitive studies (Braams et al., 2015; Urošević et al., 2012). The various studies used personality scales, temperament questionnaires, delay discounting tasks, the BAS reward responsiveness and drive scales in combination with fMRI data of various brain regions of interest. A limitation of this set of studies is that most of them cover only a limited age period: early, middle, or late adolescence and early adulthood. But the findings of these studies are quite consistent and (except for Lydon-Staley & Geier, 2018) support the curvilinear development of the socioemotional system. Socioemotional reactivity increases from early to middle adolescence and decreases thereafter. These findings strongly support the Dual Systems Model and partly the Maturational Imbalance Model.

#### Development of the balance of cognitive control and socioemotional behavior

The reviewed longitudinal studies offer some support to the Dual Systems Model and the Maturational Imbalance Model but no test of the differential predictions of both theories on the development of the balance of cognitive control and socioemotional reactivity within individuals. Both the Dual Systems Model and the Maturational Imbalance Model assume that socioemotional reactivity is stronger than cognitive control in middle adolescence. The Dual Systems Model further assumes that this mid-adolescent dominance of socioemotional reactivity over cognitive control will switch to a reversed pattern in late adolescence and early adulthood: a dominance of cognitive control over socioemotional reactivity. In contradistinction the Maturational Imbalance Model assumes that the mid-adolescent dominance of socioemotional reactivity over cognitive control will switch to a balance in late adolescence and early adulthood: a situation where both behavioral tendencies have about equal strength. To test these predictions a longitudinal person-centered approach will be adopted, as different subgroups of youth might show development corresponding with predictions of either the Dual Systems Model or the Maturational Imbalance Model.

#### Development of adolescent risk taking and links with impulse control and sensation seeking

Substance use is one of the key risk factors in adolescence and early adulthood. Recent longitudinal studies systematically report increases from early to late adolescence/early adulthood of smoking tobacco (Ashenhurst et al., 2015; Crawford et al., 2003; Lydon-Staley & Geier, 2018; Peeters et al., 2019; Quinn & Harden, 2013; Romer & Hennessy, 2007), marijuana or cannabis use (Crawford et al., 2003; Peeters et al., 2019; Quinn & Harden, 2013; Romer & Hennessy, 2007), and alcohol use (Crawford et al., 2003; Khurana et al., 2012; Peeters et al., 2019; Quinn & Harden, 2013; Romer & Hennessy, 2007). The studies suggest that substance use increases quite fast from early to middle/late adolescence and at a slower rate until age 23.

Systematic longitudinal associations (predictive links, systematic over-time correlations or parallel growth) have been found between low impulse control and high sensation seeking on the one hand and substance use on the other hand. Longitudinal associations between high impulse control (Lydon-Staley & Geier, 2018; Peeters et al., 2017) or low levels of impulsivity (Ashenhurst et al., 2015; Khurana et al., 2012; Littlefield et al., 2009; Quinn & Harden, 2013) and smoking tobacco, marijuana and alcohol use were in most studies negative. Longitudinal associations between impulse control and smoking tobacco were the least systematic. Similarly, longitudinal associations between sensation seeking and smoking tobacco (Crawford et al., 2003; Lydon-Staley & Geier, 2018; Romer & Hennessy, 2007), marijuana or cannabis use (Crawford et al., 2003; Peeters et al., 2017; Romer & Hennessy, 2007), and alcohol use (Ashenhurst et al., 2015; Crawford et al., 2003; MacPherson et al., 2010; Pedersen et al., 2012; Quinn & Harden, 2013; Romer & Hennessy, 2007) were systematically positive. In sum, these findings support the assumptions of the Dual Systems Model and the Maturational Imbalance Model on the negative association between cognitive control and adolescent risk taking and the positive one between socioemotional reactivity and risk. The findings also clarify clearly that there is a need to study the links between the balance of socioemotional reactivity and cognitive control on the one hand and substance use on the other.

## Current Study

The first aim of the present study is to test how the intra-individual balance of impulse control and sensation seeking develops from early adolescence until early adulthood. This study posits that the Dual Systems Model and the Maturational Imbalance Model have different predictions of the intra-individual development of the balance between socioemotional reactivity and cognitive control. To test this interpretation an approach is required that makes it possible to study the developmental change of the configuration of cognitive control and socioemotional reactivity within individuals as well as the heterogeneity of this developmental process. Therefore, a longitudinal person-centered approach was used: latent class growth analysis (LCGA). LCGA shows the development of the intra-individual configuration of cognitive control and socioemotional reactivity as well as the various developmental trajectories that are present in the data. This approach will immediately reveal whether the balance between socioemotional reactivity and cognitive control develops as predicted by the Dual Systems Model and the Maturational Imbalance Model. Until now this test of heterogeneity of development has not been conducted for the extended period from adolescence until early adulthood. Three hypotheses will be tested. Hypothesis 1 is derived from the Dual Systems Model and predicts that especially in middle adolescence socioemotional reactivity will be stronger than cognitive control whereas cognitive control will become stronger than socioemotional reactivity from late adolescence to early adulthood. Hypothesis 2 is derived from the Maturational Imbalance Model and also predicts that in middle adolescence socioemotional reactivity will be stronger than cognitive control whereas from late adolescence to early adulthood both behavioral tendencies will have equal strength.

The second aim is to study the longitudinal links between the various trajectories and risk behavior, that is smoking tobacco, marihuana use and alcohol use. Hypothesis 3 is that an imbalance trajectory, a trajectory with higher sensation seeking as compared to impulse control, will show higher levels of substance use than more balanced trajectories, that is, trajectories with higher levels of impulse control than sensation seeking.

## Methods

### Participants and Procedures

Data from 7,558 adolescents and early adults aged 12–25 years of age, along with data collected from their mothers were used in the current study. Data from the 1994 until 2014 waves of the National Longitudinal Study of Youth 79 (NLSY79) Children and Young Adults study (CNLSY) were included.

The data of the mothers came from the NLSY79 study initiated in 1979 by the Bureau of Labor Statistics of the US and were used to determine family SES. Details of the initial sample of the NSLY and oversampling of African American and Hispanic youth can be found in Harden and Tucker-Drob (2011). Participants have been interviewed annually since 1979 and biennially since 1994. Retention rates for the over 25 waves of data collection were between 80 and 90%.

The data from the adolescents came from the children and young adults study (CNLSY). From 1988 onward children aged over 10 years completed individual interviews and from 1994 onward children aged 15 years or older did. Informed consent was obtained from parents and children. The National Longitudinal Study of Youth 79 (NLSY79) Children and Young Adults study (CNLSY) was approved by the institutional review boards (IRBs) of Ohio State University and NORC at the University of Chicago.

The present study used data of a subsample of 7,558 adolescents (3,833 males) between the ages of 12 and 25 years who reported on impulse control and sensation seeking at least once in the CNLSY waves of 1994 until 2014. Scores of both scales were calculated only when respondents filled out at least 2 of the 3 items of the scale. The subsample is ethnically diverse: 22.1% of the adolescents were Hispanic, 32.4% were African American, and 45.5% were non-Hispanic White. Since adolescents were interviewed biennially, 2-year age groups were used to analyze the data: 12–13 year olds (T1), 14–15 year olds (T2), 16–17 year olds (T3), 18–19 year olds (T4), 20–21 year olds (T5), 22–23 year olds (T6), and 24–25 year olds (T7), summing up to 7 waves of data. Of the 7,558 adolescents used in the present study, 1,884 provided data on one time point, 4,983 on 2 until 4 time points, and 736 on 5 or more time points.

The CNLSY data overrepresent older participants who were born to relatively young mothers. To correct for this well-documented sampling bias in CNLSY family SES (a composite of family income, education, maternal cognitive ability and of maternal age at first birth, see below) that differs between younger and older mothers was controlled for in the various analyses. Additionally, also adolescent gender and ethnicity was controlled for.

### Measures

#### Impulse control and sensation seeking

Impulse control was measured with three items: (1) “I often get into a jam because I do things without thinking”; (2) “I think that planning takes the fun out of things”; and (3) “I have to use a lot of self-control to keep out of trouble”. Sensation seeking was also measured with three items: (1) “I enjoy taking risks”; (2) “I enjoy new and exciting experiences, even if they are a little frightening or unusual”; and (3) “Life with no danger in it would be too dull for me”. Items of both scales had a 4-point format ranging from 1 (strongly agree) to 4 (strongly disagree). The scale items were keyed such that higher scores indicate greater impulse control or sensation seeking. Earlier analyses have established that impulse control and sensation seeking form two distinct personality dimensions (Harden & Tucker-Drob, 2011), and also measurement invariance of both scales across the ages 10 until 25 has been established (Shulman et al., 2015). In the present study test-retest reliabilities across waves were 0.63 and 0.68 for impulse control and sensation seeking, respectively. Data coverage was 40.6 and 40.8% for impulse control and sensation seeking, respectively and Little’s Missing Completely At Random (MCAR) test produced a normed χ2 (χ2/df) of 1.35, which indicates that the data were likely missing at random, and that it is safe to impute missing values or use full information likelihood to account for them. Follow-up analyses indicated that respondents with valid T1data of impulse control and sensation seeking had only slightly different scores on T2 until T7 data of both scales as compared to respondents without T1 data: effects sizes (ή^2^) ranged between 0.000 and 0.009 or were non-significant (mean ή^2^ was 0.0026). Thus, differences between groups with and without missing data at T1 were very small.

#### Substance use

Self-reported frequencies of cigarette use and marijuana use, assessed at time points 1 until 7, were used. Cigarette and marijuana use in the last 30 days were measured with 6-point scales ranging from 0 (never) until 5 (every day). Respondents reported the frequency of drinking alcohol during the past year on a 9-point scale ranging from 1 (did not drink) to 9 (drank daily) at time points 2 until 7. For time point 1 data were not available since children aged 12 until 14 did not report on this measure of alcohol use. Little’s Missing Completely At Random (MCAR) test produced a normed χ2 (χ2/df) of 1.06, which indicates that the data were likely missing at random, and that it is safe to impute missing values in SPSS or use full information likelihood to account for them.

#### Family SES

Family income was assessed with maternal reports of total annual income, ranging between 0$ and 922.631$. Family income of the waves 1994 until 2014 was averaged. Maternal education was assessed with maternal reports of the number of years of school completed. Reports across the years 1994 until 2014 were averaged. Maternal cognitive ability was assessed by the Armed Forces Qualification Test (AFQT) in 1980. The AFQT yield composite scores on word knowledge, paragraph comprehension, math knowledge and arithmetic reasoning. The 2006 revised scores were used. Finally, maternal age at first birth was calculated using the date of birth of first child. Since these 4 indicators were moderately to strongly correlated (between 0.36 and 0.52) a confirmatory factor analysis was ran in Mplus 8.3 and showed a one factor model to fit the data well: CFI and RMSEA were 0.98 and 0.03, respectively. Factor loadings of the four variables ranged between 0.57 and 0.75. The factor was labeled as family SES, with higher and lower factor scores indicating high and low SES, respectively.

#### Ethnicity

The ethnicity of respondents was assessed by interviewer’s direct observation of the race of the mother: non-Hispanic Whites, African Americans or Hispanics.

#### Strategy of analyses

To address the first research aim and test hypotheses 1 and 2 on the presence of a Dual Systems Model and a Maturational Imbalance Model trajectory, respectively, latent class growth analysis (LCGA) was performed on impulse control and sensation seeking. An unconditional LCGA was used and five criteria were applied to determine the number of latent classes (Muthén & Muthén, 2000; Nagin, 2005). First, adding an additional class should result in improvement of model fit. A decrease of the Bayesian Information Criterion (BIC) statistic is indicative of this, as is the sample size adjusted BIC. Second, entropy, a standardized measure of classification of individuals into trajectory classes based upon the posterior probabilities of classification, should be acceptable. Entropy values range from zero to one, with values of 0.75 or higher indicating good classification accuracy (Reinecke, 2006). Third, adding an additional class should lead to an increase of fit as indicated by the bootstrapped likelihood ratio test (BLRT; Nylund et al., 2006). Fourth, the content of the classes in the various solutions was evaluated. If an additional class in a solution with k classes was found to be a slight variation of a class already found in a solution with k - 1 classes, the most parsimonious solution was chosen. Finally, every class had to cover at least 1% of the sample (see the GROLTS checklist, Van De Schoot et al., 2017). Moreover, the LCGA should be replicated with the improved BCH (Bolck, Kroon, & Hagenaars) method to correct for classification inaccuracy (Asparouhov & Muthén, 2014).

The two-class solution was found to be superior to the one-class solution and the three-class-solution superior to the two-class solution. In all comparisons, the model with more classes had a BIC and a sample size adjusted BIC that was at least 9211,34 smaller than the model with fewer classes, as well as a better fit to the data according to the BLRT (*p* < 0.0001 in all cases). Adding a fourth class did not have a surplus value, since the fourth class was found to be a variation of the second class of the three-class solution. Thus, the three-class solution was selected as the final one. Entropy (E) of this solution was good, at 0.85. The unconditional LCGA was replicated with the improved BCH (Bolck, Kroon, & Hagenaars) method to correct for classification inaccuracy (Asparouhov & Muthén, 2014) and the same three classes were found. Entropy of this model was again 0.85.

To meet the second research aim it was assessed whether the three classes of impulse control (IC) and sensation seeking (SS) showed different levels (intercepts) and change rates (slopes) of cigarette, marijuana and alcohol use. First a series of conditional latent growth models (LGM’s) was ran to determine levels and rates of change of smoking tobacco, and marijuana and alcohol use. In the LGM’s family SES, adolescent gender and ethnicity were controller for. Second, the predicted intercepts and slopes of the final LGM’s were saved for each individual. Third the growth factors of each individual were used as dependent variables in a BCH LCGA with the three IC/SS classes as groups. This procedure allows for the assessment of differences between classes membership after class membership has been estimated.

In estimating the final BCH LCGA full information maximum likelihood (Schafer & Graham, 2002) was used to account for missing data. Furthermore, standard errors and model fit statistics were adjusted for nonindependence of data from adolescents from the same family (sibling clusters, *n* = 3486). Models were estimated with a robust maximum likelihood estimation method (MLR). Family SES, adolescent gender and ethnicity were controlled for.

## Results

### Three Trajectories of Impulse Control and Sensation Seeking

**Fig. 2:**
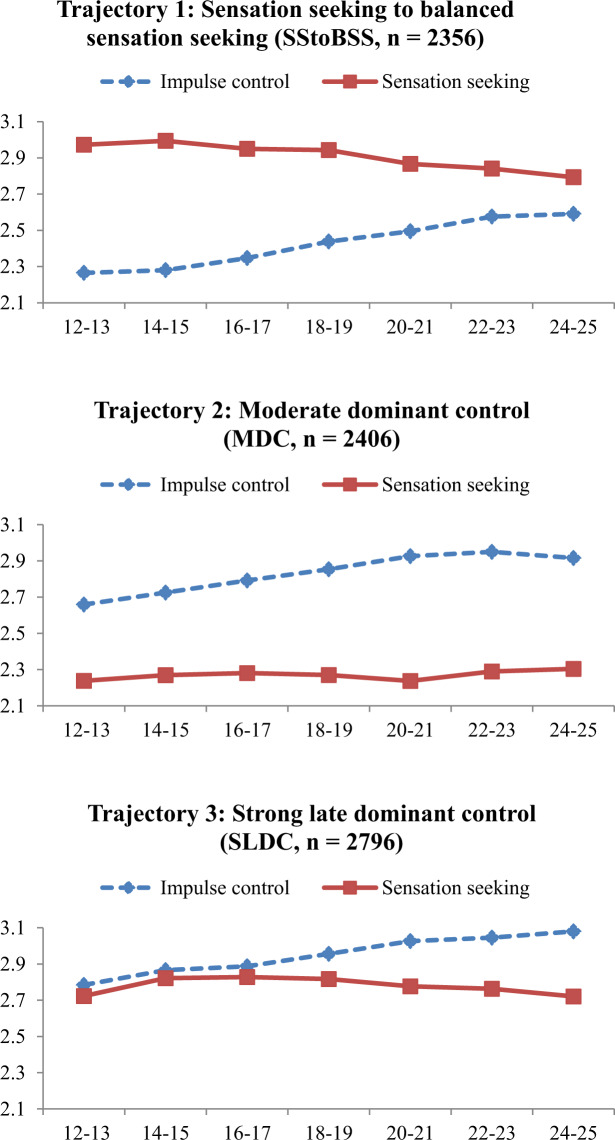
Profiles of trajectories of impulse control and sensation seeking: “sensation seeking to balanced sensation seeking” (SStoBSS), “moderate dominant control” (MDC), and “strong late dominant control” (SLDC)

The upper part of Table 1 shows the mean intercepts and slopes of the three trajectory classes. The first trajectory, “sensation seeking to balanced sensation seeking” (SStoBSS) showed a higher level of sensation seeking than impulse control across all ages. The dominance of sensation seeking over impulse control, however, became significantly smaller when adolescents age. The second trajectory, “moderate dominant control” (MDC), showed moderate and increasing impulse control that overrode sensation seeking across all ages. The third trajectory, “strong late dominant control” (SLDC), showed the highest level of impulse control of the trajectories which was about as strong as sensation seeking from early until middle adolescence and became substantially stronger from late adolescence to early adulthood. Figure 2 offers a graphical representation of impulse control and sensation seeking within the three trajectories. Two additional findings of the three-class solution were of interest: linear growth of impulse control and a (small) curvilinear development of sensation seeking were found in all classes. In the first and the third class the curvilinear trend indicated somewhat higher levels of sensation seeking in the middle part of adolescence, whereas the trend in the second class indicated a somewhat lower level at age 20–21.

**Table 1:** Trajectories of impulse control and sensation seeking and between-trajectory differences in substance use. Findings of the final BCH model note

Parameter estimates (means)	IC/SS trajectories		
	Sensation seeking to balanced sensation seeking (SStoBSS)	Moderate dominant control (MDC)	Strong late dominant control (SLDC)
	Growth factors		
Impulse control			
- Intercept	2.23***	2.64***	2.78***
- Linear slope	0.07***	0.11***	0.07***
- Quadratic slope	−0.001	−0.009***	−0.003***
Sensation seeking			
- Intercept	2.99***	2.27***	2.76***
- Linear slope	−0.01	−0.008	0.04***
- Quadratic slope	−0.004***	0.002**	−0.009***

	Differences between IC/SS trajectories in (growth of) substance use		
Smoking tobacco			
- Intercept	0.13^a^	0.09^b^	0.05^c^
- Linear slope	0.74^a^	0.44^b^	0.45^b^
- Quadratic slope	−0.06^a^	−0.03^c^	−0.04^b^
Marijuana use			
- Intercept	0.04^a^	0.03^b^	0.02^c^
- Linear slope	0.37^a^	0.13^b^	0.14^b^
- Quadratic slope	−0.03^a^	−0.009^b^	−0.007^b^
Alcohol use			
- Intercept	1.40^a^	1.03^c^	1.23^b^
- Linear slope	1.19^b^	0.93^c^	1.25^a^
- Quadratic slope	−0.10^a^	−0.06^b^	−0.10^a^

Means with different superscripts across columns indicate between trajectory differences in substance use at *p* ≤ 0.001

**p < 0.01, ***p < 0.001

These findings do not support hypotheses 1 and 2. Hypothesis 1 was derived from the Dual Systems Model and predicted that especially in middle adolescence sensation seeking would be stronger than impulse control whereas impulse control would become stronger than sensation seeking from late adolescence to early adulthood. Hypothesis 2, derived from the Maturational Imbalance Model, also predicted that in middle adolescence sensation seeking would be stronger than impulse control whereas from late adolescence on both behavioral tendencies would have equal strength. None of the three trajectories showed the Dual Systems Model or the Maturational Imbalance Model pattern. The strong late dominant control (SLDC) trajectory supported the Dual Systems Model somewhat since impulse control indeed continued to increase and became stronger than sensation seeking from late adolescence to early adulthood. Similarly, the sensation seeking to balanced sensation seeking (SStoBSS) trajectory supported the Maturational Imbalance Model somewhat since sensation seeking and impulse control became more balanced from late adolescence to early adulthood.

### Trajectories of Impulse Control and Sensation Seeking and Adolescent Substance Use

The second aim was to study the longitudinal links between the development of the various IC/SS trajectories and risk behavior. Results are presented in 3 steps. First, the development of adolescent smoking tobacco, marijuana use and alcohol use was modeled. Second, a BCH model was conducted to demonstrate the differences in substance use between the various IC/SS trajectories.

#### Development of adolescent substance use

A series of conditional growth curve models (LGM’s) was estimated across 7 time points (ages 12–13, 14–15, 16–17, 18–19, 20–21, 22–23 and 24–25) for adolescent smoking tobacco, marijuana use and across 6 time points (from ages 14–15 on) for alcohol use. Three criteria were used to compare linear with nonlinear growth models: a significant Satorra-Bentler scaled χ^2^ difference test (Steiger et al., 1985), a difference in CFI > 0.01 (Cheung & Rensvold, 2002), and a difference in RMSEA > 0.01 (Chen 2007). For all three study variables (i.e., smoking tobacco, drinking and marijuana use) quadratic models provided a significantly better fit with the data compared to the linear models. ∆S-B χ^2^ (7)’s, ΔCFI’s, and ΔRMSEA’s of the nonlinear versus the linear models of smoking tobacco, marijuana use and alcohol were at least 530.60 (all *p*’s < 0.001), 0.11 and 0.02, respectively. The nonlinear models produced good fit for each of the substances: CFI’s ≥ 0.94 and RMSEA’s ≤ 0.04, respectively. For each substance growth was relatively fast from early to middle/late adolescence and slowed somewhat down thereafter. The growth factors of the three nonlinear models were saved for each individual for use in the final BCH model.

#### IC/SS trajectories and substance use

Hypothesis 3 stems from both the Dual Systems Model and the Maturational Imbalance Model. Both models predict that dominance of sensation seeking over impulse control goes together with higher risk. Therefore, the “sensation seeking to balanced sensation seeking” (SStoBSS) trajectory should reveal elevated levels of substance use as compared to the “moderate dominant control” (MDC) and “strong late dominant control” (SLDC) trajectories where impulse control is expected to override sensation seeking. To test hypothesis 3 a BCH LCGA was conducted with the three trajectories (classes) as predictors of the individual growth factors of substance use. To account for differences in background characteristics of IC/SS types gender of adolescent, family SES and ethnic group were included as covariates. Additionally, the difference in substance use between the moderate dominant control (MDC) and strong late dominant control (SLDC) trajectories was explored. Since these trajectories were not predicted by the Dual Systems Model and the Maturational Imbalance Model it was not possible to formulate hypotheses on differences in substance use between them. Entropy of the BCH LCGA was very good: 0.85. Table 1, lower part, shows the results.

**Fig. 3:**
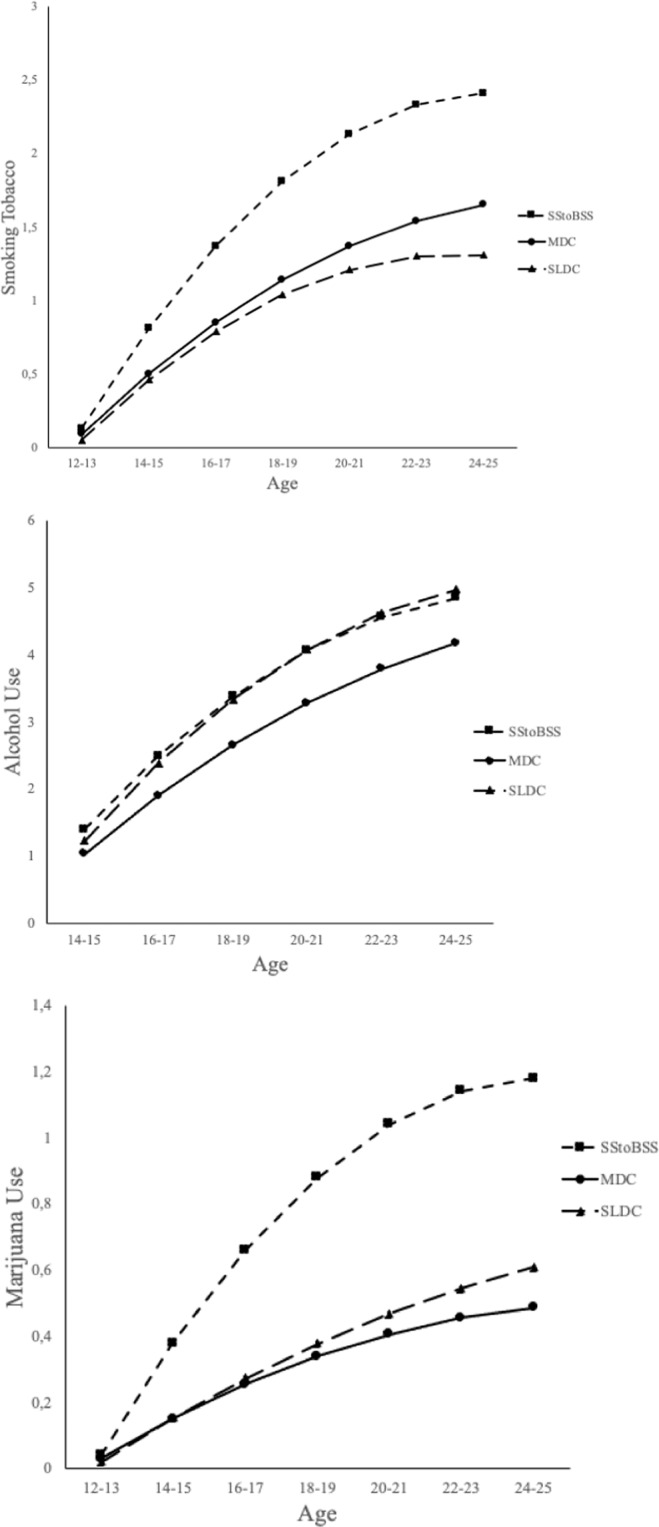
Between-trajectory differences in substance use. SStoBSS is “sensation seeking to balanced sensation seeking”; MDC is “moderate dominant control”; SLDC is “strong late dominant control”

Hypothesis 3 is supported. The “sensation seeking to balanced sensation seeking” (SStoBSS) trajectory showed the highest levels and smoking tobacco, marijuana and alcohol use, as well as the biggest linear growth of smoking tobacco and marijuana use, see Fig. 3. The “sensation seeking to balanced sensation seeking” (SStoBSS) trajectory also showed the highest quadratic slopes indicating that the growth of substance use shows a somewhat stronger decelerating trend as compared to the other trajectories. Therefore, the “sensation seeking to balanced sensation seeking” (SStoBSS) trajectory showed the highest risk of substance use. There was one exception to this rule: the increase of alcohol use was stronger in the “strong late dominant control” (SLDC) than in the in the “sensation seeking to balanced sensation seeking” (SStoBSS) trajectory (see Fig. 3). These findings provide support to both the Dual Systems Model and the Maturational Imbalance Model, given that individuals with an imbalance between impulse control and sensation seeking showed the highest risk of substance use. In addition, Table 1 reveals differences between the “moderate dominant control” (MDC) and “strong late dominant control” (SLDC) trajectories. The “moderate dominant control” (MDC) trajectory showed higher levels and linear growth of smoking tobacco and marijuana use as compared to the “strong late dominant control” (SLDC) trajectory, whereas the SLDC trajectory showed a higher level and linear growth of alcohol use.

To check for the robustness of the findings, hypothesis 3 was also tested for the non-abstainers, that is to say, the group that smoked, used marijuana or drank. Since the number of non-abstainers was different for each of the substances three separate BCH LCGA’s were conducted. Support for hypothesis 3 was found for each of the substances. All three BCH LCGA’s showed that the “sensation seeking to balanced sensation seeking” (SStoBSS) trajectory had higher intercepts (all substances) and slopes (with the exception of drinking) as compared to the “moderate dominant control” (MDC) and “strong late dominant control” (SLDC) trajectories. Individuals in the “sensation seeking to balanced sensation seeking” (SStoBSS) trajectory started substance use earlier and showed stronger growth than the “moderate dominant control” (MDC) and “strong late dominant control” (SLDC) trajectories in smoking tobacco and marijuana use. Again, there was one exception to this rule: the increase of alcohol use was stronger in the “strong late dominant control” (SLDC) than in the in the “sensation seeking to balanced sensation seeking” (SStoBSS) trajectory. In conclusion, support was found for hypothesis 3 in the whole sample, that is abstainers and non-abstainers and in the group that was comprised of active substance users: the non-abstainers. The findings of the three BCH LCGA’s can be found in Table S1 of the supplementary material.

## Discussion

The Dual Systems Model and the Maturational Imbalance Model are heuristic models to explain adolescent risk. In the present study a person-centered approach was used to conceptualize the Dual Systems Model and the Maturational Imbalance Model as theories of intra-individual development of cognitive control and socioemotional reactivity. The first hypothesis was derived from the Dual Systems Model, which predicted that sensation seeking would be stronger than impulse control, especially in middle adolescence, whereas impulse control would become stronger than sensation seeking from late adolescence into adulthood. The second hypothesis, which was derived from the Maturational Imbalance Model, predicted that in middle adolescence sensation seeking would be stronger than impulse control whereas from late adolescence on both behavioral tendencies would have equal strength. Both hypotheses were not supported: none of the three observed trajectories of impulse control and sensation seeking showed the pattern predicted by the Dual Systems Model or the Maturational Imbalance Model. Some support was found for the Dual Systems Model in the strong late dominant control (SLDC) trajectory: impulse control indeed became stronger than sensation seeking from late adolescence to early adulthood. Similarly, the “sensation seeking to balanced sensation seeking” (SStoBSS) trajectory supported the Maturational Imbalance Model somewhat since sensation seeking and impulse control became more balanced from late adolescence to early adulthood, although at very high levels of sensation seeking.

Hypothesis 3 was derived from both the Dual Systems Model and the Maturational Imbalance Model and predicted that dominance of sensation seeking over impulse control would go together with high risk. Support for this hypothesis was indeed found: the trajectory with dominance of sensation seeking over impulse control, the “sensation seeking to balanced sensation seeking” (SStoBSS) trajectory, showed the highest levels of substance use. It should be noted that this finding could be attributed to the high levels of sensation seeking and low levels of control. In sum the present study could not provide strong support for the predictions of the Dual Systems Model and the Maturational Imbalance Model on the intra-individual development of cognitive control and socioemotional reactivity but provided support for their predictions regarding adolescent risk taking. The results underline the importance of high levels of sensation seeking as a risk factor for substance use, in particular when combined with low levels of self-control. However, the results do not confirm the assumed age-related peak in this imbalance in middle adolescence as explanation for (the assumed peak in) high levels of substance use.

### Imbalance of Cognitive Control and Socioemotional Reactivity and Risk Taking

Although the Dual Systems Model and the Maturational Imbalance Model generally describe developmental changes in impulse control and sensation seeking in all adolescents, the results of the current study showed that there is substantial heterogeneity in this development, such that different groups of adolescents show a differential development in the balance between these two systems. The findings show that a substantial group of adolescents and early adults experiences a dominance of sensation seeking over impulse control: 31.2% belong to the “sensation seeking to balanced sensation seeking” (SStoBSS) trajectory. This means that about one third of youth shows imbalance of sensation seeking and impulse control. The imbalance tends to diminish as adolescents get older due to growing impulse control in particular. Thus, the assumed imbalance is not a general phenomenon in adolescence or early adulthood but a profile that is characteristic for a––substantial––minority of this age group.

The findings also make clear that sensation seeking only showed the expected U-shaped curve in the “strong late dominant control” trajectory (SLDC), which included 37% of the sample. The peak in sensation seeking in this trajectory was present in early and middle adolescence and seemed to be consistent with the peak of delinquency in middle adolescence (see Meeus, 2016, for an overview). The analyses of the present paper, however, showed that patterns that were suggested to be present in a whole group (the U-shaped curve) are actually only found for a subgroup. This makes clear that it is useful to study heterogeneity of developmental processes. It is also important to note that over 60% of the respondents showed impulse control that overrides sensation seeking (the “moderate dominant control” (MDC) and the “strong late dominant control” (SLDC) trajectories). This result underscores that most of the adolescents and early adults are capable to control their impulses.

The Dual Systems Model and the Maturational Imbalance Model are accurate in predicting the link between (im)balance and risk taking. Two key findings of this study support this conclusion. (1) The “sensation seeking to balanced sensation seeking” (SStoBSS) trajectory showed the highest levels of risk behavior: smoking tobacco, marijuana and alcohol use. (2) The more balanced “moderate dominant control” (MDC) and “strong late dominant control” (SLDC) trajectories showed lower risk. There is no evidence that decreasing imbalance in the “sensation seeking to balanced sensation seeking” (SStoBSS) trajectory goes together with a decrease or lower increase in risk. Additionally, the results suggest that imbalance may have lasting detrimental effects. As the growth factors in Table 1 demonstrate, the “sensation seeking to balanced sensation seeking” (SStoBSS) trajectory maintains elevated levels of substance use at age 25. Although the imbalance in this group has become smaller by then, its detrimental effects are still present. The higher risk in the “sensation seeking to balanced sensation seeking” (SStoBSS) trajectory as compared to the two other trajectories during adolescence and early adulthood is due to the higher scores on sensation seeking and the lower scores on impulse control relative to the other two groups. It should also be noted that the higher risk in the “sensation seeking to balanced sensation seeking” (SStoBSS) trajectory as compared to the two other trajectories during adolescence and early adulthood may result from the fact that while the imbalance in the “sensation seeking to balanced sensation seeking” (SStoBSS) trajectory decreases, the balance in the two other trajectories increases. The dominance of impulse control over sensation seeking increases in the “moderate dominant control” (MDC) and “strong late dominant control” (SLDC) trajectories. These findings also suggest that imbalance goes together with risk in adolescence and early adulthood and counter the critique that dual systems models provide no evidence on the link between imbalance and real-world risk (Meisel et al., 2019). In addition, it should be noted that links between processes of balance and imbalance and risk taking were found after controlling for gender, family SES and ethnicity. This shows that these links occur independent of background characteristics of respondents.

The findings underscore the role of individual differences in impulse control and sensation seeking. In all three IC/SS trajectories the vast majority of respondents showed a quite stable patterns of dominance of sensation seeking over impulse control across ages (the sensation seeking to balanced sensation seeking (SStoBSS) trajectory) or the reversed pattern (the moderate dominant control (MDC) and strong late dominant control (SLDC) trajectories). Only in early to middle adolescents in the ‘strong late dominant control” (SLDC) trajectory this dominance of one behavioral tendency over the other is less consistent. Therefore, individual differences in impulse control and sensation seeking should not be overlooked in the explanation of risk behavior. This interpretation links to the suggestion by Bjork and Pardini (2015) who stated that the high-risk type is not a developmental type but represents individuals with quite stable disruptive behavior disorder (DBD).

### On Neuroscience and Developmental Research

Steinberg’s Dual Systems Model is a social neuroscience model and Casey’s Maturational Imbalance Model a neurobiological model. In the interpretation of the present paper both models offer theories that conceptualize adolescent risk taking as due to the imbalance of two neurobiological systems: relative early development of subcortical socioemotional reactivity in combination with relative late development of the prefrontal cognitive control system. As shown above the findings do not offer strong support for key developmental assumptions of both models but it should be noted that the present study did not use neurobiological measures. The measures of impulse control and sensation seeking are proxies of neurobiological processes of cognitive control and socioemotional reactivity. This calls for a replication and extension of the present study including both behavioral and neurobiological measures (see also Becht & Mills, 2020).

The present study informs us on the requirements of neurobiological studies on the development of (im)balance in adolescents and early adults. These requirements concern the design, measurements and analytical approach of such a study (see also vol. 33 of Developmental Cognitive Neuroscience: Methodological Challenges in Developmental Neuroimaging: Contemporary Approaches and Solutions). (1) An important next step for neurobiological studies is to rely less on drawing developmental conclusions from age comparisons of cross-sectional studies (a limitation also noted by Meisel et al., 2019) and adopt longitudinal designs that cover the age range from early adolescence until early adulthood and include a substantial sample size. (2) Measures need to tap relatively stable individual differences, that are differences that are stable between individuals across situations. Differences between participants that occur only in very specific test situations have limitations because they probably have weak over time rank order stability. Rank order stability is a requirement to study systematic development: measures that are not predictive of themselves have a low test-retest reliability and therefore are less suitable to study regular developmental processes. This implies that stronger rank order stabilities are conditional for the appearance of regular developmental trajectories. The present study convincingly shows this phenomenon: three regular developmental trajectories were found and this pattern of findings is due to the strong rank order stabilities of impulse control and sensation seeking that were present. Across time points 1–2, 2–3, 3–4, 4–5, 5–6, and 6–7 they were 0.40, 0.54, 0.60, 0.74, 0.75, 0.77 and 0.49, 0.60, 0.66, 0.67, 0.87, 0.77 for impulse control and sensation seeking, respectively. (3) Analytical approaches should address (im)balance of cognitive control and socioemotional reactivity within persons. This requires analysis of the development of the configuration of these two processes within individuals and person-centered developmental approaches such as LCGA (see for a further discussion of this issue Meisel et al., 2019).

### Limitations of the present study

The present study has a number of limitations. A first limitation is that it cannot be claimed that imbalance of cognitive control and socioemotional reactivity causes risk taking. That would require a (quasi-) experimental approach. Second, the current study had a very strong longitudinal design in terms of behavioral measures but did not include experimental of brain imaging measures. This calls for a replication and extension of the present study with additional measures. The sample size of such a study should be large enough to study heterogeneity of development of cognitive control and socioemotional reactivity. Third: the measures of impulse control and sensation seeking were very short. A replication with more nuanced measures is warranted.

## Conclusion

This study was designed to scrutinize developmental heterogeneity of cognitive control and socioemotional reactivity. This developmental heterogeneity was indeed found. This is a critical contribution since it uncovers nuanced developmental patterns of cognitive control and socioemotional reactivity that are not visible in whole-sample analyses. Therefore it can be concluded that the Dual Systems Model and the Maturational Imbalance Model do not offer an accurate account of the development of the (im)balance of cognitive control and socioemotional reactivity in adolescence and early adulthood. None of the three observed trajectories of impulse control and sensation seeking showed the pattern predicted by the Dual Systems Model or the Maturational Imbalance Model. However, some support was found for the Dual Systems Model in the “strong late dominant control” (SLDC) trajectory: impulse control indeed became stronger than sensation seeking from late adolescence to early adulthood. Similarly, the “sensation seeking to balanced sensation seeking” (SStoBSS) trajectory supported the Maturational Imbalance Model since sensation seeking and impulse control became more balanced from late adolescence to adulthood. Moreover, support was found for a key assumption of both theories, namely that imbalance, conceptualized as dominance of sensation seeking over impulse control, goes together with risk taking, as measured with substance abuse, in adolescence and early adulthood. It was observed that 31.2% of adolescents is at risk for elevated substance use and that this risk extends beyond adolescence into early adulthood. Finally, it was also found that most adolescents (68.8%) grow into relatively high impulse control and low sensation seeking (the “moderate dominant control” (MDC) and “strong late dominant control” (SLDC) trajectories). This again adds to the prior theories showing that most young people are able to meet the challenges of adolescence.

## Supplementary information

Supplementary Table S1
