# Supplementary material for: On Imbalance of Impulse Control and Sensation Seeking and Adolescent Risk: An Intra-individual Developmental Test of the Dual Systems and Maturational Imbalance Models
Source: J Youth Adolesc. 2021 Mar 20;50(5):827–40. doi: 10.1007/s10964-021-01419-x (PMC8043917; doi:10.1007/s10964-021-01419-x)
Supplement: Supplementary file 1 — Supplementary Table S1 [file 10964_2021_1419_MOESM1_ESM.docx]

Table S1

*Between-Trajectory Differences in Substance Use in Non-abstainers. Findings of the three BCH Models*

| Parameter estimates (Means) | IC/SS trajectories | | |
| --- | --- | --- | --- |
|  | Sensation seeking to balanced sensation seeking (SStoBSS) | Moderate dominant control (MDC) | Strong late dominant control (SLDC) |
|  | Differences between IC/SS trajectories in (growth of) substance use | | |
| Smoking tobacco   - Intercept - Linear slope - Quadratic slope | *n* = 2201  .15^a^  .74^a^  -.06^a^ | *n =* 2239  .10^b^  .46^c^  -.03^c^ | *n =* 2526  .05^c^  .49^b^  -.04^b^ |
| Marijuana use   - Intercept - Linear slope - Quadratic slope | *n =* 1620  .06^a^  .47^a^  not estimated^1^ | *n =* 1685  .04^b^  .16^c^  not estimated^1^ | *n =* 1565  .04^b^  .24^b^  not estimated^1^ |
| Alcohol use   - Intercept - Linear slope - Quadratic slope | *n =* 1926  1.58^a^  1.21^b^  -.11^a^ | *n =* 1943  1.28^c^  1.00^c^  -.08^b^ | *n =* 2461  1.46^b^  1.29^a^  -.11^a^ |

*Note.* Means with different superscripts across columns indicate between trajectory differences in substance use at *p* ≤ .001.

^1^ Due to estimation problems the quadratic slope could not be calculated.

** *p* < .01 *** *p* < .001
